# Supplementary material for: Synthesis of Yttria-Stabilized Zirconia Nanospheres from Zirconium-Based Metal–Organic Frameworks and the Dielectric Properties
Source: Nanomaterials (Basel). 2022 Dec 21;13(1):28. doi: 10.3390/nano13010028 (PMC9824511; doi:10.3390/nano13010028)
Supplement: Supplementary file 1 [file nanomaterials-13-00028-s001.zip › nanomaterials-2093657-supplementary.pdf]

nanomaterials

*Supplementary Materials*

# Synthesis of Yttria-Stabilized Zirconia Nanospheres from Zirconium-Based Metal–Organic Frameworks and the Dielectric Properties

**Hyun Woo Park [1,2,†], Eunyeong Cho [1,3,†], Yun Zou [4,5,†], Sea Hoon Lee [4,5], Jae Ryung Choi [1], Sang-Bok Lee [1], Kyeongwoon Chung [6], Se Hun Kwon [2,*], Jeonghun Kim [3,*] and Hee Jung Lee [1,*]**

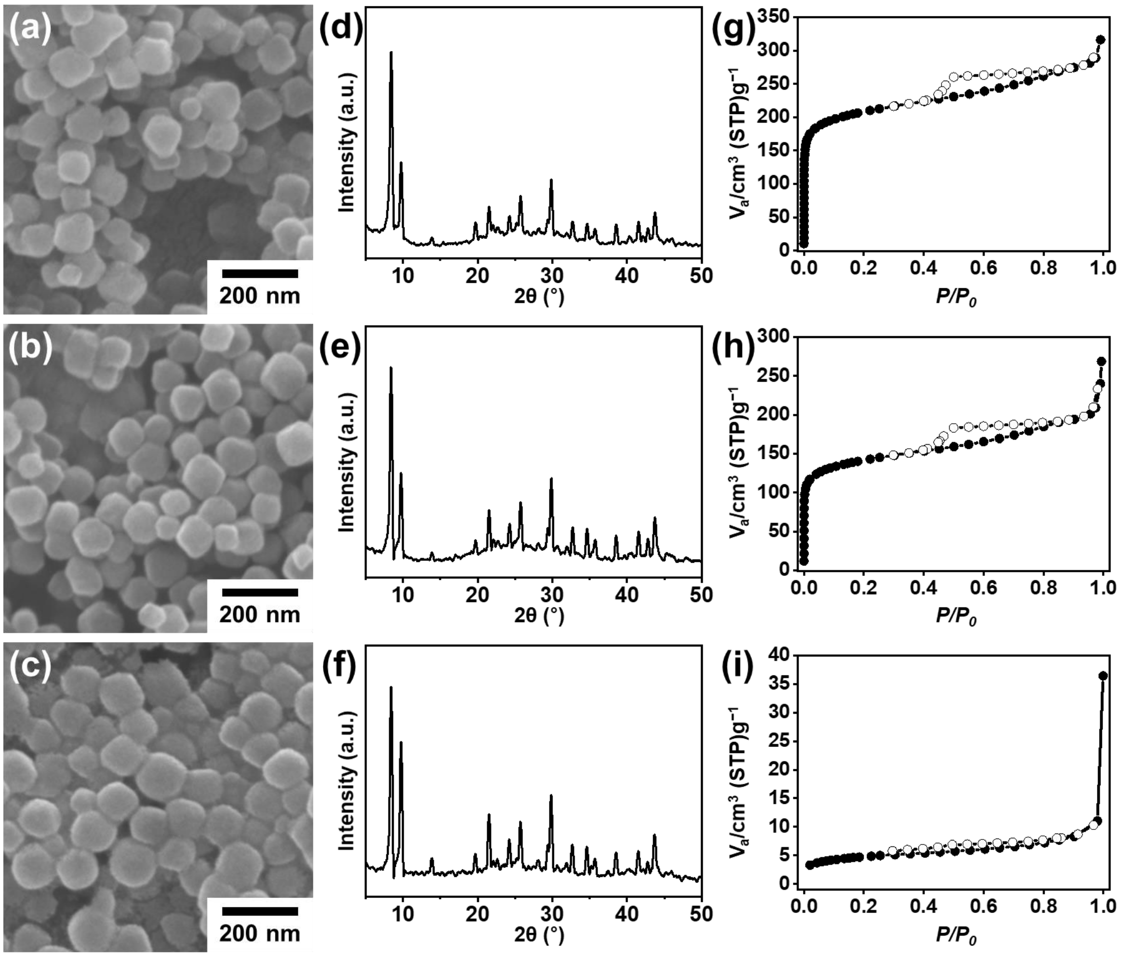


**Figure S1.** FE−SEM images, XRD patterns, and nitrogen adsorption/desorption isotherms of the YSZ precursors: (**a**,**d**,**g**) 3YSZ, (**b**,**e**,**h**) 8YSZ, and (**c**,**f**,**i**) 30YSZ.

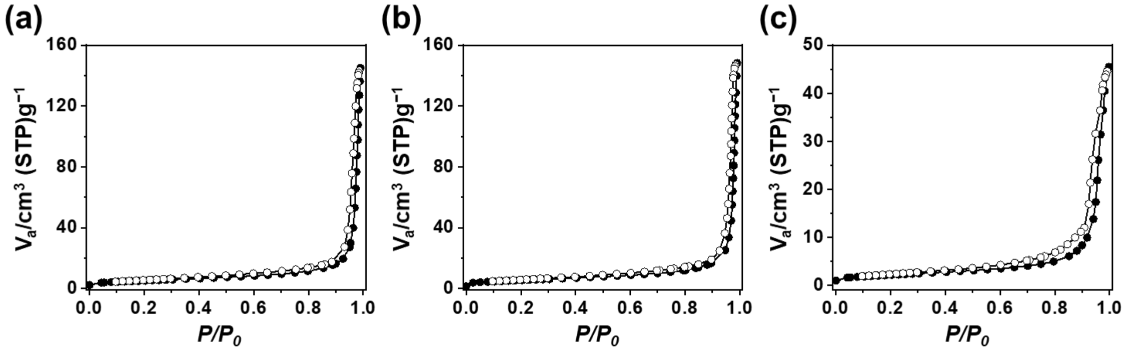


**Figure S2.** Nitrogen adsorption/desorption isotherms of the YSZ nanospheres: (**a**) 3YSZ, (**b**) 8YSZ, and (**c**) 30YSZ.

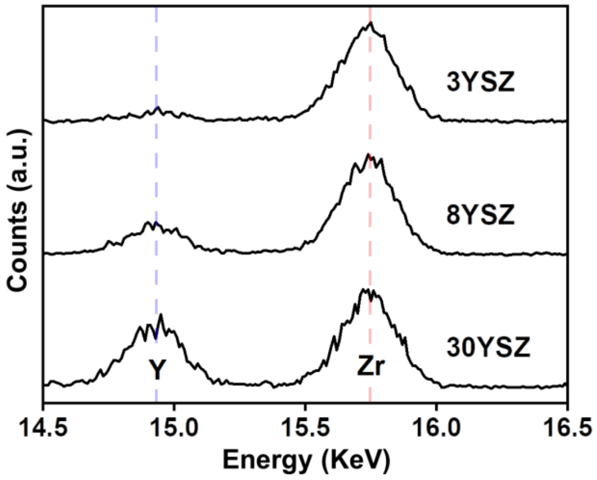


**Figure S3.** EDX spectra of the 3YSZ (top), 8YSZ (middle), and 30YSZ (bottom) nanospheres.

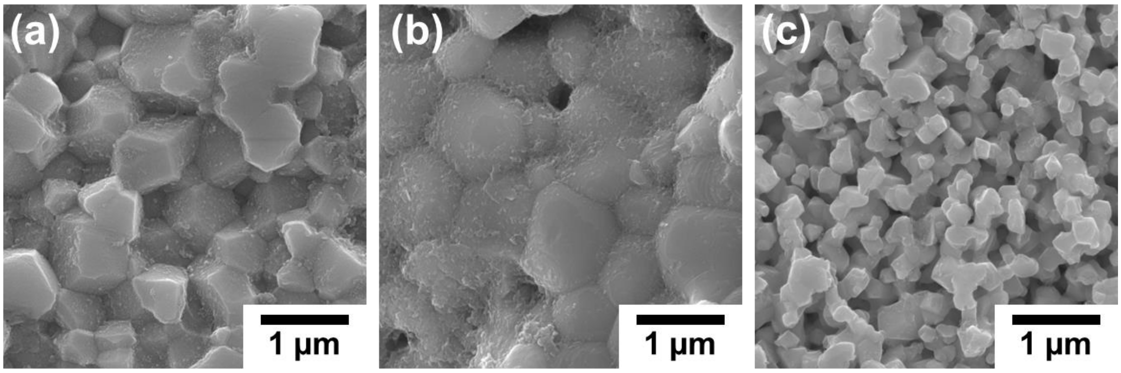


**Figure S4.** SEM images of the sintered YSZ bodies: (**a**) 3YSZ, (**b**) 8YSZ, and (**c**) 30YSZ.

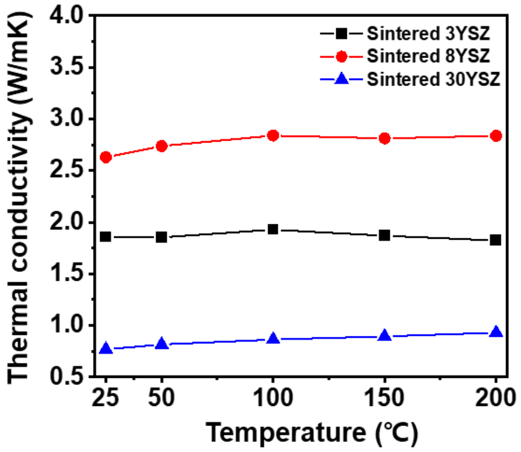


**Figure S5.** Thermal conductivity of the sintered YSZ bodies with various temperatures.
